# Identification of the epigenetic reader CBX2 as a potential drug target in advanced prostate cancer

**DOI:** 10.1186/s13148-016-0182-9

**Published:** 2016-02-12

**Authors:** Pier-Luc Clermont, Francesco Crea, Yan Ting Chiang, Dong Lin, Amy Zhang, James Z. L. Wang, Abhijit Parolia, Rebecca Wu, Hui Xue, Yuwei Wang, Jiarui Ding, Kelsie L. Thu, Wan L. Lam, Sohrab P. Shah, Colin C. Collins, Yuzhuo Wang, Cheryl D. Helgason

**Affiliations:** Department of Experimental Therapeutics, British Columbia Cancer Research Centre, 675 W 10th Avenue, Vancouver, British Columbia V5Z 1L3 Canada; Faculty of Medicine, MD Program, Université Laval, 1050, avenue de la Médecine, Québec, QC G1V 0A6 Canada; Vancouver Prostate Centre, 899 West 12th Avenue, Vancouver, British Columbia V5Z 1M9 Canada; Department of Life, Health, and Chemical Sciences, The Open University, Milton Keynes, MK7 6BH UK; Department of Computer Science, Faculty of Science, University of British Columbia, 2366 Main Mall, Vancouver, British Columbia V6T 1Z4 Canada; Department of Molecular Oncology, British Columbia Cancer Research Centre, 675 W 10th Avenue, Vancouver, British Columbia V5Z 1L3 Canada; Genetics Unit, Department of Integrative Oncology, British Columbia Cancer Research Centre, 675 W 10th Avenue, Vancouver, British Columbia V5Z 1L3 Canada; Department of Urologic Sciences, Faculty of Medicine, University of British Columbia, 2775 Laurel Street, Vancouver, British Columbia V5Z 1M9 Canada; Department of Surgery, University of British Columbia, 910 W 10th Avenue, Vancouver, British Columbia V5Z 4E3 Canada

**Keywords:** Castration-resistant prostate cancer, CBX2, Epigenetics, Metastatic prostate cancer, Polycomb

## Abstract

**Background:**

While localized prostate cancer (PCa) can be effectively cured, metastatic disease inevitably progresses to a lethal state called castration-resistant prostate cancer (CRPC). Emerging evidence suggests that aberrant epigenetic repression by the polycomb group (PcG) complexes fuels PCa progression, providing novel therapeutic opportunities.

**Results:**

In the search for potential epigenetic drivers of CRPC, we analyzed the molecular profile of PcG members in patient-derived xenografts and clinical samples. Overall, our results identify the PcG protein and methyl-lysine reader CBX2 as a potential therapeutic target in advanced PCa. We report that CBX2 was recurrently up-regulated in metastatic CRPC and that elevated CBX2 expression was correlated with poor clinical outcome in PCa cohorts. Furthermore, CBX2 depletion abrogated cell viability and induced caspase 3-mediated apoptosis in metastatic PCa cell lines. Mechanistically explaining this phenotype, microarray analysis in CBX2-depleted cells revealed that CBX2 controls the expression of many key regulators of cell proliferation and metastasis.

**Conclusions:**

Taken together, this study provides the first evidence that CBX2 inhibition induces cancer cell death, positioning CBX2 as an attractive drug target in lethal CRPC.

## Background

At present, prostate cancer (PCa) represents the most commonly diagnosed non-cutaneous malignancy in men [1]. While localized disease can be effectively treated with surgery or radiotherapy, metastatic PCa remains invariably fatal [2]. For the past 30 years, androgen-deprivation therapy (ADT) has been the standard care for disseminated PCa. However, all tumors eventually acquire resistance to ADT and relapse in a highly aggressive state called castration-resistant prostate cancer (CRPC) [3]. Despite the introduction of novel therapeutic agents for late-stage patients, CRPC remains an incurable malignancy and thus a better understanding of its molecular drivers is required to facilitate the development of novel treatment strategies [4, 5]. Over the past decade, mounting evidence has demonstrated that epigenetic alterations significantly contribute to PCa progression, suggesting that the PCa epigenome may harbor clinically relevant therapeutic targets [6].

Epigenetics refers to changes in transcriptional programs that cannot be attributed to modifications in DNA sequence [7]. Epigenetic changes result in cellular and physiological phenotypic trait variations in response to external or environmental factors that switch genes on and off. Epigenetic regulation influences gene expression by controlling access of the transcriptional machinery to distinct genomic regions [8]. During embryonic development, epigenetic mechanisms define gene expression programs which themselves specify differentiation into distinct tissues [9]. In human cancers, these epigenetic states become disrupted, thereby promoting disease initiation and progression by altering the expression of key oncogenes and tumor suppressors [10, 11]. Given the clinical approval of a growing number of epigenetic drugs, there is considerable value in identifying novel chromatin-regulating complexes driving disease progression [12].

Emerging evidence suggests that epigenetic dysregulation mediated by the polycomb group (PcG) family of transcriptional repressors plays a critical role during PCa progression [13]. Conserved throughout evolution, PcG proteins assemble in two main polycomb repressive complexes, PRC1 and PRC2 [14]. In the classical model, PRC2 trimethylates histone H3 at lysine 27 (H3K27me3) via the catalytic activity of EZH2, thereby triggering transcriptional silencing [15]. H3K27me3 can then be recognized by the N-terminal chromodomain of five CBX proteins (CBX2, 4, 6, 7, 8), which are members of PRC1 [16]. Upon binding H3K27me3, CBX proteins can recruit PRC1 to chromatin through protein-protein interactions. PRC1 recruitment further promotes transcriptional repression through various mechanisms such as histone H2A ubiquitination and chromatin compaction, some of which are known to play a role in PCa progression [17, 18]. In advanced PCa, EZH2 is overexpressed and pharmacological inhibition of PRC2 impairs tumorigenicity and metastatic ability [13, 19]. Moreover, the PRC1 member BMI1 promotes resistance to docetaxel, a drug used in CRPC treatment via modulation of key transcriptomic programs [20]. While the tumor-promoting roles of EZH2 and BMI1 have been well established, the functional implication of individual PcG members during PCa progression and their contribution to CRPC have yet to be evaluated.

Since CBX proteins bridge the activity of PRC2 and PRC1, they represent critical regulators of PcG-mediated silencing [21]. We have previously demonstrated that CBX2 expression was significantly up-regulated in aggressive tumors of many cancer types, including PCa [22]. These novel findings complement studies from *CBX2*-deficient animals demonstrating critical functions for CBX2 in cellular proliferation and differentiation [23, 24]. It has been shown that animal models lacking *CBX2* display multi-organ hypocellularity as a result of a proliferative block. In mice, germline deletion of the *CBX2* homolog *M33* results in homeotic transformations and sexual defects [25, 26]. Strikingly, it was shown across multiple species that individuals with XY karyotype lacking *CBX2* were unable to undergo development of the male urogenital system, implying a role in prostatic cell proliferation and differentiation [26, 27]. Taken together, these findings indicate that CBX2 may be functionally involved in aberrant PcG-mediated silencing thought to promote PCa progression and drug resistance.

With the aim of identifying new epigenetic targets, we analyzed the molecular profiles of PcG family members in patient-derived xenograft (PDX) models and clinical samples of advanced PCa. Using validated in vitro and in vivo models [28, 29], we demonstrate that the PRC1 member and epigenetic reader CBX2 is recurrently overexpressed in metastatic and androgen-independent PCa cells and that elevated CBX2 expression predicts poor clinical outcome. Furthermore, we show that CBX2 depletion induces PCa cell death and proliferation arrest by regulating the expression of a key subset of genes, suggesting that CBX2 may emerge as a novel therapeutic target for advanced PCa.

## Results

### CBX2 is overexpressed in aggressive PCa

**Fig. 1:**
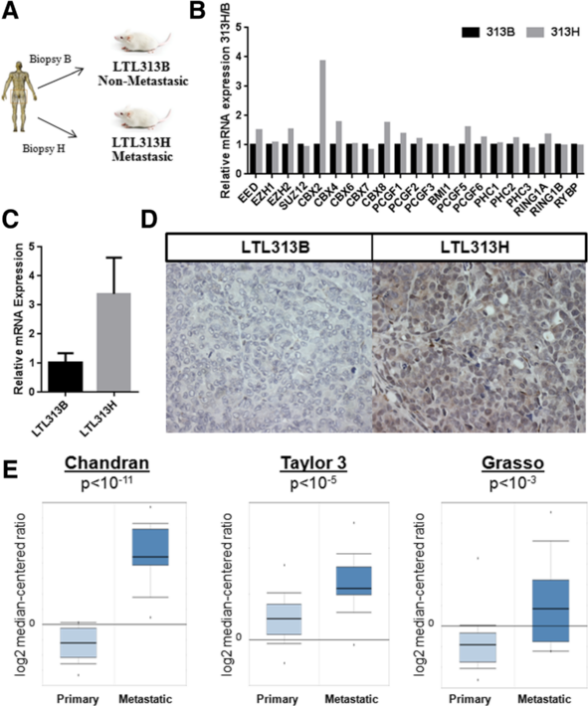
CBX2 is overexpressed in metastatic PCa. **a** Establishment of the LTL313B/LTL313H PDX model of metastatic PCa; **b** Expression of core PcG family members in the LTL313H/LTL313B xenograft model; Results are based on a single microarray experiment; **c** Confirmation of CBX2 mRNA up-regulation in the LTL313H tumor line by qRT-PCR; **d** Confirmation of CBX2 protein up-regulation in the LTL313H tumor line by IHC (20x). Images are representative of multiple fields taken from 2 independent experiments; **e** Elevated CBX2 mRNA levels in metastatic PCa compared to non-metastatic samples in three independent patients

As the first step to identify putative therapeutic targets for advanced PCa, we analyzed the expression of PcG genes in the LTL313H/LTL313B PDX model of metastatic and non-metastatic PCa [29]. LTL313H and LTL313B represent two xenografted tissues that were derived from two independent needle biopsies of the same primary PCa tumor (Fig. 1a). This unique PDX pair therefore recapitulates and exploits the intra-tumoral heterogeneity observed in clinical PCa as LTL313H consistently gives rises to metastases when implanted in the mouse subrenal capsule while LTL313B always stays local to the grafting site. Interestingly, genomic characterization has previously determined that the genetic profile of LTL313B and LTL313H displays more than 95 % homology [29], implying that epigenetic alterations are likely to be involved in the process of metastatic dissemination. Thus, this model provides a unique experimental system to identify differential expression of PcG genes between distinct *foci* of different metastatic ability within a single primary prostate tumor [29].

Microarray analysis was performed on RNA extracted from LTL313B and LTL313H to identify differential expression of PcG genes. This analysis demonstrated that the chromodomain-containing protein, and known regulator of male urogenital system development, CBX2, was the most highly up-regulated PcG transcript in LTL313H compared to LTL313B (Fig. 1b). To validate these results, we assessed CBX2 expression in both tumor lines using quantitative reverse transcription polymerase chain reaction (qRT-PCR), which confirmed that CBX2 expression was 3.2-fold higher in LTL313H compared to LTL313B (Fig. 1c, *p* < 0.0001, Student’s *t* test). Consistent with messenger RNA (mRNA) levels, CBX2 protein expression was undetectable in LTL313B while LTL313H showed strong CBX2 immunostaining, in line with a possible role in PCa dissemination (Fig. 1d, ×20).

To ensure that overexpression of CBX2 in metastatic PCa tissues was not solely a property of the LTL313B/LTL313H xenograft model, we assessed the expression of CBX2 in primary and metastatic tumors from PCa patients using the Oncomine database [30]. As observed in the xenografts, CBX2 expression was significantly higher in metastatic compared to non-metastatic tumors in three independent clinical cohorts (Fig. 1e, *p* ≤ 0.05, Student’s *t* test). Importantly, we could not find a single study in which CBX2 was significantly down-regulated in metastatic tissues. Thus, the CBX2 up-regulation observed in the LTL313B/LTL313H PDX model was also recapitulated in patient tumors.

After observing elevated CBX2 levels in advanced PCa models, we sought to determine whether CBX2 overexpression correlated with specific indicators of poor outcome. We conducted multivariate analysis of variance (MANOVA) to associate the expression of CBX2 with specific clinicopathologic features in clinical PCa patients using previously published clinical data [31]. This analysis revealed that elevated CBX2 levels were significantly correlated with lower patient age, higher Gleason grade, and a positive nodal status (Table 1, *p* < 0.05, MANOVA). All these variables are themselves indicators of poor prognosis in patients; these data support the idea that elevated CBX2 expression is observed in aggressive prostate tumors.

**Table 1:** Multivariate analysis of variance correlating CBX2 and clinicopathological features in primary PCa from MSKCC cohort

Factor	*F* value	*p* value	Significance
Age	4.8235	0.030674	*
Extension	1.9261	0.131084	
Gleason	5.5086	0.021142	*
Nodal	15.4775	0.000165	***
Race	0.7067	0.55053	
Sem Vesicle	0.0262	0.871665	
SurgMargins	0.0839	0.772712	
T stage	0.5005	0.607944	

*FDR* false discovery rate

***p ≤ 0.001; *p ≤ 0.05

### Hormonal regulation of CBX2 expression

**Fig. 2:**
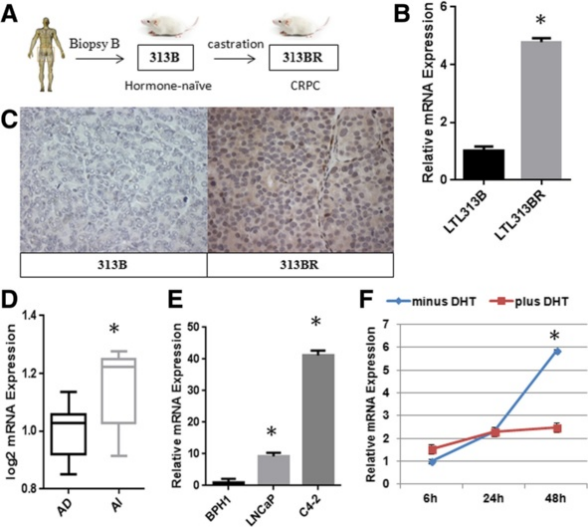
Hormonal regulation of CBX2. **a** Establishment of the LTL313B/LTL313BR patient-derived xenograft model of CRPC; **b** Assessment of CBX2 mRNA levels in the LTL313B/LTL313BR xenograft model by qRT-PCR; **c** IHC staining of CBX2 in the LTL313B and LTL313BR xenografts ×20. Images are representative of multiple fields taken from two independent experiments; **d** Levels of CBX2 mRNA in androgen-dependent (AD, *n* = 10) and androgen-independent (AI, *n* = 5) PDXs from the LTL; **e** Relative CBX2 expression in PCa cell lines compared to benign control (BPH1) assessed by qRT-PCR; **f** CBX2 mRNA levels in LNCaP cells cultured in charcoaled-stripped media in the presence or absence of DHT supplementation (10 nM)

Since metastatic PCa patients inevitably develop lethal CRPC [3], we investigated the involvement of CBX2 in the progression to androgen-independent disease. To address this question, we took advantage of another patient-derived xenograft model in which the primary tumor line, LTL313B, was subjected to ADT (Fig. 2a) [29]. As observed in the clinic, ADT elicited a significant reduction in LTL313B tumor volume shortly after castration. However, the tumor developed resistance and eventually re-emerged as the CRPC tumor line LTL313BR [29]. LTL313BR retains important properties of CRPC such as expression of PSA and androgen-independent growth, as well as resistance to AR antagonists and docetaxel [29]. Additional information regarding this model is available at the Living Tumor Laboratory website (www.livingtumorlab.com).

As the first step to link CBX2 and CRPC pathogenesis, we quantified the expression of CBX2 in the LTL313B/LTL313BR xenograft model and observed that CBX2 expression was elevated in LTL313BR relative to LTL313B using qRT-PCR (Fig. 2b, *p* < 0.001, Student’s *t* test). Furthermore, immunohistochemical (IHC) staining revealed that CBX2 protein levels were undetectable in LTL313B while LTL313BR exhibited strong CBX2 nuclear staining (Fig. 2c). To confirm the results obtained in the 313B/BR model, we assessed the expression of CBX2 in a panel of PCa PDX models that were either androgen-dependent (*n* = 10) or androgen-independent (*n* = 5) available at the Living Tumor Laboratory. In line with the 313B/BR model, CBX2 expression was significantly higher in the androgen-independent PDX models (Fig. 2d, *p* < 0.05, Student’s *t* test), consistent with a role in castration-resistant disease.

To complement the observations made in PDX models, we conducted in vitro studies investigating the androgenic regulation of CBX2. First, we quantified the expression of CBX2 in LNCaP and C4-2 cell lines compared with benign prostate hyperplasia cells (BPH1). The isogenic LNCaP/C4-2 model was chosen for these studies since it represents a validated and clinically relevant model of PCa progression. LNCaP was originally derived from a lymph node metastasis. It was subsequently implanted into a castrated mouse, giving rise to a castrate-resistant cell line C4-2 following ADT [32]. Both LNCaP and C4-2 express AR, but only LNCaP exhibits androgen-responsive growth [32]. Moreover, C4-2 xenografts display higher tumor formation and produce more metastatic foci in vivo, consistent with the idea that androgen-independent cells are inherently more aggressive [28]. Androgen-independent C4-2 cells displayed CBX2 mRNA levels 41 times higher than BPH1 while androgen-dependent LNCaP exhibited a nine-fold up-regulation in CBX2 expression (Fig. 2e, *p* < 0.0001 for both, Student’s *t* test). Next, CBX2 expression was assessed in vitro using androgen-responsive LNCaP cells subjected to removal and addition of dihydrotestosterone (DHT), a potent AR agonist. In LNCaP cells, CBX2 mRNA levels significantly increased after 48 h of culture in androgen-depleted media, as assessed by qRT-PCR (Fig. 2f, *p* < 0.001, Student’s *t* test). Accordingly, this dramatic effect was not observed in cells supplemented with DHT, suggesting that a decrease in ligand-induced AR transactivation reversibly stimulates CBX2 expression.

Given the elevated expression of CBX2 in PCa, we set out to determine whether any genetic aberrations could be underlying CBX2 up-regulation. We queried four independent patient cohorts for which both copy number changes and mutations were available. A striking observation was that not a single point mutation could be found within the *CBX2* locus in any of the four datasets, which were comprised of a total of 329 patients (Table 2). Additionally, only 3 out of 329 patients (0.9 %) were found to have a CBX2 copy number loss (CNL). Similarly, only 5 out of 329 patients (1.5 %) exhibited CBX2 copy number gain, which is not sufficient to account for the CBX2 up-regulation observed in clinical PCa (Table 2). Taken together, these findings highlight the rarity of genomic disruption of CBX2 and suggest that CBX2 itself is likely to be under epigenetic and/or hormonal regulation.

**Table 2:** Genomic alterations affecting the CBX2 locus in PCa

PCa dataset and journal	No. of patients	% Mut	% CNG	% CNL
MSKCC—Cancer Cell 2010	103	0	2	1
Michigan—Nature 2012	61	0	5	0
Broad/Cornell—Nat. Gen. 2012	109	0	0	0
Broad/Cornell—Cell 2013	56	0	0	4
Total	329	0	2	1

### CBX2 depletion induces cell death in advanced PCa cell lines

**Fig. 3:**
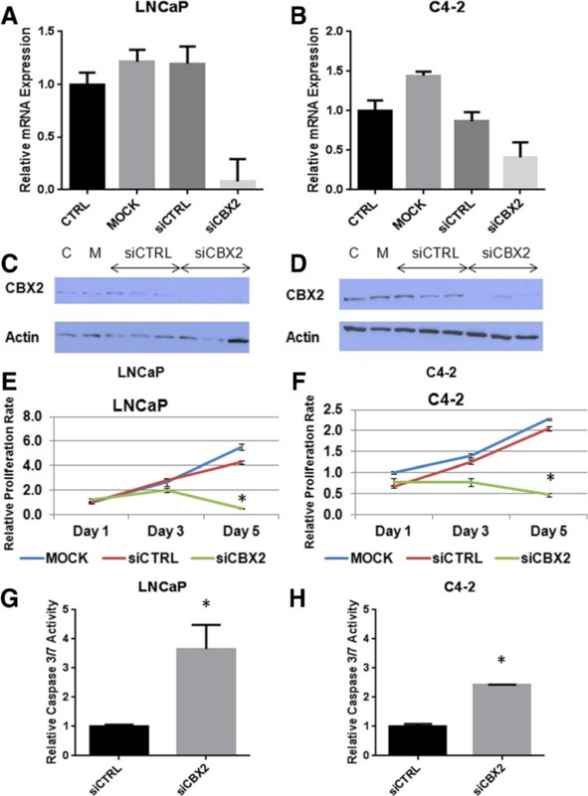
CBX2 depletion induces proliferation arrest and apoptosis in advanced PCa cell lines. **a**, **b** Confirmation of CBX2 mRNA knockdown in LNCaP and C4-2 cells by qPCR; **c**, **d** Confirmation of CBX2 protein knockdown in LNCaP and C4-2 cells; **e**, **f** MTT analysis of cell viability following CBX2 silencing in LNCaP and C4-2 cells; **g**, **h** Assessment of caspase 3–7 activity in LNCaP and C4-2 cells following CBX2 depletion

**Fig. 4:**
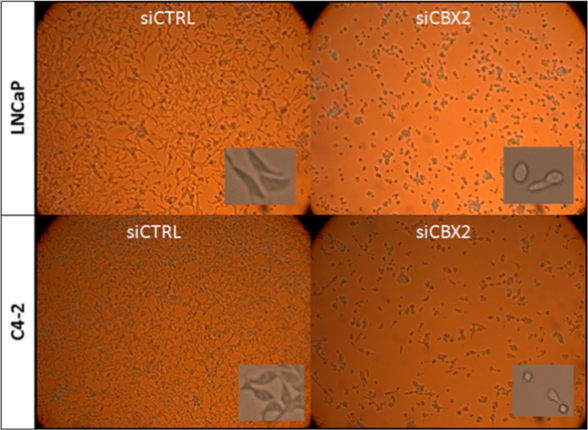
Morphology of LNCaP and C4-2 cells following CBX2 depletion (96 h post-siRNA treatment). Images are representative of multiple fields taken from three independent experiments (×20 for large image and ×40 for small image)

To evaluate the functional requirements of CBX2 in advanced PCa cells, we analyzed the phenotypic effects of small interfering RNA (siRNA)-mediated CBX2 silencing in two metastatic PCa cell lines, LNCaP and C4-2. In LNCaP cells, both CBX2 mRNA and protein levels were reduced by more than 90 % following siRNA treatment (Fig. 3a, c, *p* < 0.0001, Student’s *t* test). For C4-2 cells, CBX2-specific siRNA induced a 60 % reduction in CBX2 mRNA levels while CBX2 protein levels were variably reduced (Fig. 3b, d, *p* < 0.0001, Student’s *t* test). Approximately 55 h following transfection, both LNCaP and C4-2 cells treated with CBX2-specific siRNA started exhibiting notable morphological changes not observed in cells treated with non-targeting siRNA. In both the LNCaP and C4-2 lines, cells started to round up and lose their epithelial appearance (Fig. 4). As these morphological changes occurred, the cells stopped proliferating and started detaching from the plate after about 3 days post-transfection, leaving very few viable cells 4 days after siRNA treatment.

To quantify the extent of cell viability loss resulting from CBX2 depletion, we conducted 3-(4,5-dimethylthiazol-2-yl)-2,5-diphenyltetrazolium bromide (MTT) analysis on LNCaP and C4-2 cells treated with mock, non-targeting control, or CBX2-specific siRNA. MTT assay confirmed a significant reduction in cell viability following CBX2 knockdown in both cell lines (Fig. 3e, f, *p* < 0.0001, Student’s *t* test). More specifically, the proliferation arrest induced by CBX2 depletion started to appear 3 days after siRNA treatment and culminated in a dramatic decrease in cell viability after 5 days in both cell lines, thus confirming the microscopic observations. To explore the possibility that CBX2 might regulate apoptotic cell death, caspase 3/7 activity was analyzed in LNCaP and C4-2 cells treated with either control or CBX2-specific siRNA for 72 h. Notably, CBX2 depletion induced a 3.7- and 2.3-fold increase in caspase 3/7 activity in LNCaP and C4-2, respectively (Fig. 3g, h, *p* < 0.001, Student’s *t* test), suggesting that CBX2 is required for PCa cell survival. Taken together, these findings indicate that CBX2 is functionally involved in the regulation of PCa cell morphology, proliferation, and apoptosis.

### Gene expression profiling of CBX2-depleted cells

**Fig. 5:**
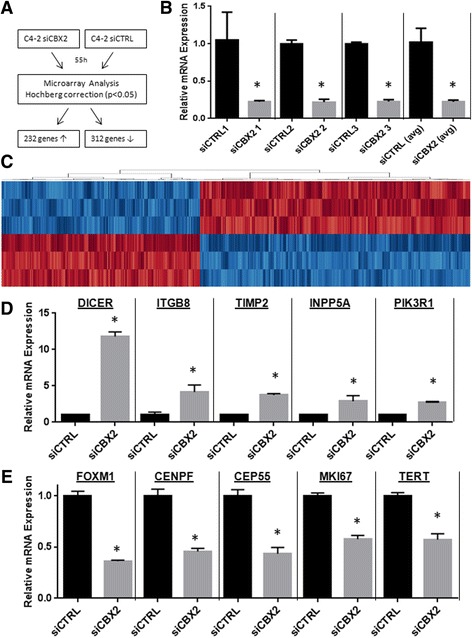
Gene expression profiling of CBX2-regulated genes. **a** Experimental design of microarray analysis; **b** Validation of CBX2 silencing in samples subjected to microarray analysis; **c** Unsupervised hierarchical clustering of genes differentially expressed following CBX2 knockdown; **d** Differential expression of up-regulated CRGs confirmed by qRT-PCR in CBX2-depleted C4-2 cells; **e** Differential expression of down-regulated CRGs confirmed by qRT-PCR in CBX2-depleted C4-2 cells

Given the striking phenotypes observed upon CBX2 depletion, we further investigated the molecular mechanisms and transcriptomic changes controlled by CBX2 in CRPC. To identify CBX2-regulated genes (CRGs), we conducted microarray profiling in the CRPC cell line model C4-2 treated with control or CBX2-specific siRNA (Fig. 5a). RNA was extracted 55 h after siRNA transfection, a time point where CBX2 expression is reduced in siCBX2-treated cells but just prior to when these cells start to display abnormal proliferation and morphology (Fig. 3). Three replicate samples were obtained for each condition to ensure reproducibility. To validate optimal RNA quality, we assessed the purity and integrity of the RNA via Nanodrop and Bioanalyzer, respectively. Nanodrop analysis revealed that all replicates had A_280_/A_230_ and A_260_/A_230_ ratios higher than 2.0, indicating high RNA purity. In addition, Bioanalyzer studies demonstrated that all six samples had an RIN value higher than 9.4 out of 10 (average = 9.65), indicating high quality and minimal degradation across all replicates.

After validating RNA quality and knockdown efficiency, we conducted microarray analysis using the Agilent platform. First, we conducted qRT-PCR and validated an 80 % inhibition of CBX2 expression in cells treated with CBX2-specific siRNA (Fig. 5b, *p* < 0.0001, Student’s *t* test). Using an unpaired *t* test with Benjamini-Hochberg correction, we identified 544 transcripts that were differentially expressed upon CBX2 silencing and were termed CBX2-regulated genes (CRGs, Fig. 5a). Among them, 232 were up-regulated and 312 were down-regulated (Fig. 5a). Unsupervised hierarchical clustering revealed that the up-regulated and down-regulated genes have distinct expression patterns which are extremely consistent across all replicates (Fig. 5c).

To ensure that the expression changes observed in the microarray profiling were reproducible, we first selected individual CRGs previously associated with cancer whose expression could be validated by qRT-PCR. Interestingly, a number of important regulators of cell proliferation and metastasis were significantly modulated after CBX2 depletion. Notably, ITGB8, DICER1, INPP5A, PIK3R1, and TIMP2 are key tumor suppressors that were among up-regulated CRGs following CBX2 knockdown. Significant up-regulation of these genes in CBX2-depleted cells was also validated using qRT-PCR (Fig. 5d, *p* ≤ 0.05 for all, Student’s *t* test). Conversely, the tumor-associated proteins MKI67, FOXM1, CENPF, TERT, and CEP55 were down-regulated following CBX2 silencing, which was also successfully confirmed by qRT-PCR (Fig. 5e, *p* ≤ 0.05 for all, Student’s *t* test). Thus, qRT-PCR replicated the transcriptomic changes detected through microarray analysis, providing another quality control to ensure the validity of the microarray results.

### Biological properties of CBX2-regulated genes

As the first step to analyzing the properties of CRGs, we assessed whether CRGs were associated with human diseases using Ingenuity Pathway Analysis (IPA) software. Interestingly, we found that cancer was the disease most significantly linked to CRGs (Table 3), in line with our previous finding that CBX2 is involved in a wide range of cancer types [22]. Moreover, other diseases most associated with CRGs included “Developmental Disorder” and “Reproductive System Disease,” both of which have previously been linked to CBX2 mutations in the medical literature [27]. Next, we assessed the biological properties associated with CRGs. Using the Oncomine software set at the analysis of “biological processes and functions,” a significant link between CBX2 and cell cycle progression was observed. Out of the top 13 processes most significantly correlated with CRGs, 11 were directly involved in the regulation of cell cycle progression (Table 4, inclusion criteria: odds ratio (OR) > 2, *p* < 0.05). These included “DNA replication and chromosome cycle,” “Mitotic chromosome condensation,” and “Mitotic sister chromatid segregation” (Table 4, all *p* < 0.001, all OR > 23). Thus, pathway analysis revealed that CRGs were enriched in genes involved in the control of cellular proliferation.

**Table 3:** Top diseases associated with CBX2-regulated genes (IPA analysis)

Rank	Category	*p* value
1	Cancer	5.86E-10–1.71E-02
2	Development disorder	1.60E-08–1.70E-02
3	Hematological disease	1.60E-08–1.03E-02
4	Hereditary disorder	1.60E-08–1.70E-02
5	Gastrointestinal disease	3.55E-08–8.34E-03
6	Reproductive system disease	4.46E-08–1.70E-02

**Table 4:** Biological processes associated with CBX2-regulated genes (Oncomine analysis)

Rank	Concept name	*p* value	*Q* value	Odds ratio
1	DNA replication and chromosome cycle	2.3E-06	1.2E-04	39.0
2	Mitotic chromosome condensation	8.2E-04	2.4E-02	23.2
3	Mitotic sister chromatid segregation	8.2E-04	2.4E-02	23.2
4	G1/S transition of mitotic cell cycle	1.0E-02	1.8E-01	7.7
5	Nucleotide-excision repair	1.0E-02	1.8E-01	7.7
6	Mitosis	1.5E-06	7.9E-05	6.5
7	DNA repair	4.7E-08	3.1E-06	6.3
8	DNA replication	2.7E-06	1.4E-04	6.1
9	Cytokinesis	7.2E-07	4.1E-05	5.8
10	Chromosome organization and biogenesis	1.0E-03	4.0E-02	4.6
11	Cell Cycle	2.3E-05	1.0E-03	3.9
12	Regulation of cell cycle	4.0E-03	8.0E-02	2.6
13	Intracellular signaling cascade	6.1E-04	1.9E-02	2.4

Since biological processes associated with mitosis were overrepresented in CRGs, we analyzed the expression of key genes involved in cell division. A striking feature was that several key components of the mitotic machinery were also significantly down-regulated upon CBX2 silencing. These genes encoded numerous members of the following group of mitotic proteins: centromere proteins (CENPA, E, H, I, K, L, N, O, P, Q, W), kinesin family (KIF22, 23), spindle and kinetochore associated complex subunit (SKA1, 2, 3), and structural maintenance of chromosomes (SMC2, 4) (Table 5, all *p* < 0.05, unpaired *t* test). A number of additional mitotic signaling proteins such as AURKA, AURKB, CCNB1, MKI67, CDK1, and CDC25A were also significantly down-regulated (Table 5, all *p* < 0.05, unpaired *t* test). Interestingly, the expression of the PLK family of kinases (PLK1, 3, 4) was also repressed upon CBX2 silencing (Table 5, all *p* < 0.05, unpaired *t* test). The inability to undergo mitosis caused by widespread down-regulation of proteins involved in mitotic integrity could therefore partly explain the strong proliferative defect induced by CBX2 knockdown.

**Table 5:** Expression of CBX2-regulated genes involved in mitosis following CBX2 silencing

Gene	Fold change	*p* value	Gene	Fold change	*p* value
CENP family			SMC family		
CENPA	−3.1	2.1E-03	SMC1	−2.0	3.3E-02
CENPE	−3.0	1.6E-04	SMC2	−2.7	1.4E-04
CENPH	−2.9	4.4E-03	SMC3	−1.3	3.4E-03
CENPI	−3.0	1.7E-03	SMC4	−2.9	1.1E-03
CENPK	−2.4	1.0E-03	SMC6	−1.5	3.0E-03
CENPL	−1.8	5.6E-03	Mitotic signaling proteins		
CENPN	−1.9	1.1E-04	AURKA	−2.6	3.7E-04
CENPO	−2.4	4.2E-03	AURKB	−3.4	1.3E-03
CENPP	−1.6	2.4E-02	CCNB1	−2.4	5.0E-04
CENPQ	−1.8	1.1E-03	KI67	−2.0	1.2E-04
CENPW	−2.8	1.1E-03	CDK1	−2.3	2.2E-04
SKA family			CDC25A	2.2	6.7E-04
SKA1	−3.0	1.4E-04	PLK1	−2.7	1.0E-02
SKA2	−2.1	4.9E-03	PLK3	−1.4	4.8E-05
SKA3	−2.8	3.5E-05	PLK4	−2.9	1.5E-03

### Clinical analysis of CBX2-regulated genes

**Fig. 6:**
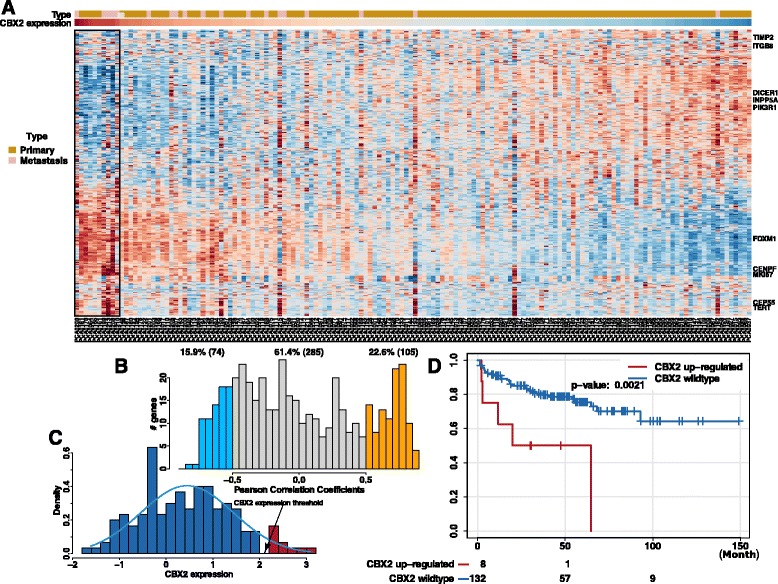
Clinical analysis of CBX2 and CBX2-regulated genes in the MSKCC prostate adenocarcinoma cohort. **a** Heatmap showing the expression of the 544 genes differentially expressed after knocking-down CBX2. Here, only the 140 patients with gene expression data are shown. The columns (patients) were sorted based on CBX2 expression (*red*: high expression, *blue*: low expression). Metastatic prostate cancer patients had significantly higher CBX2; **b** CBX2 expression correlated with the expression of the differently expressed genes; **c** CBX2 expression distribution across the 140 patients. Here, we used a CBX2 expression threshold of 2 to call CBX2 up-regulation since there was a natural gap around expression value of 2; **d** Patients with CBX2 expression up-regulation had significantly lower disease-free survival

To determine whether gene expression changes observed upon CBX2 silencing had clinical relevance, we analyzed the expression of CRGs in a large clinical dataset containing both primary and metastatic tumors [31]. First, we sorted patients based on their CBX2 mRNA expression. In line with our previous findings, metastatic PCa had significantly higher CBX2 expression compared to primary PCa (Fig. 6a, *p* < 0.0001, Mann-Whitney *U* test). Next, we performed Ward’s clustering to observe the distribution of CRGs based on CBX2 expression. The resulting heatmap clearly demonstrated that a large proportion of CRGs show apparent clustering, indicating that CRGs are correlated with CBX2 expression. To quantify the relationship between CBX2 and individual CRGs in patient tumors, we calculated the Pearson correlation coefficient (*ρ*) between CBX2 expression and expression of each CRG across patients. As expected, the expression of a number of CRGs was strongly correlated with CBX2 expression (i.e., *ρ* higher than 0.5 or *ρ* lower than −0.5). More specifically, 75 genes (15.9 %) had a *ρ* lower than 0.5, and 105 (22.6 %) had a *ρ* lower than −0.5. These findings confirm that the CRGs found upon CBX2 silencing in vitro (see Fig. 4) are also correlated with CBX2 expression in patient tumors, suggesting that CBX2 is the causative agent behind clinical gene expression programs.

Finally, we determined whether CBX2 expression had an impact on clinical outcome. We first created a density plot demonstrating the spectrum of CBX2 expression in PCa. Since there was a natural cutoff at CBX2 expression around 2, we separated patients based on this cutoff and performed logrank test (Fig. 6c). Analysis of the resulting Kaplan-Meier curve indicated that patients with higher CBX2 expression displayed a significantly lower disease-free survival compared to patients with lower CBX2 levels (Fig. 6d, *p* = 0.0021, logrank test). Taken together, these findings demonstrate that CBX2 expression correlates with specific gene expression programs in patients and is associated with poor clinical outcome.

## Discussion

Despite numerous large-scale sequencing efforts, very few genetic mutations are recurrently found in PCa, suggesting that epigenetic alterations likely contribute to PCa progression [33]. Recent studies have highlighted a critical role for the PcG family of epigenetic repressors in PCa cell survival and metastasis [17]. We therefore analyzed the expression of all PcG members in paired primary/metastatic PDXs and clinical datasets of PCa. Our results demonstrate that CBX2 is the most highly up-regulated PcG member across multiple models of metastatic and castration-resistant PCa and that elevated CBX2 levels correlate with poor clinical outcome. Moreover, we show for the first time that CBX2 depletion induced PCa cell death in vitro, which was accompanied by differential expression of key genes regulating PCa progression. Taken together, these results position CBX2 as a putative therapeutic target in advanced PCa.

CBX2 up-regulation was first identified in our paired non-metastatic (LTL313B) and metastatic (LTL313H) PDXs implanted into the subrenal capsule of NOD-SCID mice [29]. We have previously shown that this type of PDX conserves the molecular profile of the parental patient tumor. A particular feature of the LTL313B/H model is that both tumor lines originate from different *foci* of a single localized tumor, thus properly recapitulating the intra-tumoral heterogeneity observed in clinical PCa [29]. In the LTL313B/H model, we observed a high expression of CBX2 solely in the metastatic tumor line LTL313H. Based on this model, our results suggest that a small population of CBX2-expressing PCa cells within the primary tumor is the likely seed of metastatic dissemination. Consistent with this notion, we have also shown that CBX2 expression is elevated in metastatic tumors compared to those remaining local to the prostate. This is in accordance with our in vitro studies, which demonstrate that CBX2 depletion induced death in two metastatic PCa cell lines. Further supporting this idea, CBX2 inhibition resulted in up-regulation of PI3K antagonists such as PIK3R1 and INPP5A. In turn, this would result in activation of the pro-metastatic PI3K/AKT pathway, which is known to be altered in the vast majority of CRPC patients [34].

Currently, a major clinical challenge lies in identifying patients who will develop lethal, disseminated PCa and those who will not progress to metastatic disease [35]. Given the strong association between CBX2 and aggressive PCa, the expression of CBX2 could provide prognostic information. We found that elevated CBX2 levels independently predicted high grade, metastatic dissemination, and disease-free survival in PCa patients. However, as observed in the LTL313B/H model, there exists intra-tumoral heterogeneity within primary PCa such that molecular analyses resulting from a single biopsy site may not detect all CBX2-overexpressing *foci*. Therefore, we propose that positive CBX2 IHC staining in at least one core biopsy could be incorporated as an unfavorable prognostic marker that could be interpreted in the context of currently used methods such as TNM staging and Gleason score.

In line with the idea that CBX2 promotes tumor progression, the biological processes and functions associated with CRGs were intricately related with proliferation. These properties are consistent with phenotypic features of CBX2-deficient animals which exhibit multi-organ hypocellularity as a result of a proliferative block [25]. Further linking CBX2 and cell cycle progression, the analysis of CRGs revealed that a large number of proteins involved in mitotic spindle assembly are significantly down-regulated upon CBX2 silencing. In the literature, there is evidence demonstrating that CBX2 directly contributes to cell cycle progression through its association with condensed chromatin [36, 37]. Here, we expand on this mitotic function and show that, in addition, CBX2 also ensures integrity of cell division indirectly via the regulation of CRGs involved in mitotic spindle assembly. Moreover, CRGs included targetable kinases of the aurora kinase (AURKA, B) and the polo-like kinase (PLK1, 3, 4) families, all of which have been shown to promote G2/M transition. Taken together, these results suggest that CBX2 represents a key regulator of mitosis in CRPC, in line with its reported role in cellular proliferation.

A striking phenotype of *CBX2*-KO animals and humans is that XY subjects undergo male-to-female reversal, implying that CBX2 is required for the development of the male urogenital system [27]. While this feature suggests that CBX2 may cooperate with AR activity, our data indicates that CBX2 is antagonistically regulated by ligand-dependent AR signaling. Given the pro-survival properties conferred by CBX2 in vitro, we posit that CBX2 up-regulation may serve as an adaptive mechanism to bypass the anti-tumor response elicited by castration. Currently, an emerging clinical problem is that CRPC patients are becoming increasingly susceptible to transdifferentiation into highly aggressive neuroendocrine prostate cancer (NEPC) as a result of treatment with novel AR suppressors [38]. We have recently demonstrated that a number of PcG genes including CBX2 were overexpressed in NEPC [39]. Given the up-regulation of CBX2 in both CRPC and NEPC, we posit that CBX2 is required for tumor cell survival following castration but that other molecular mechanisms define specialization into neuroendocrine or epithelial lineages. As a consequence, development of CBX2 antagonists may benefit patients with late-stage disease by simultaneously blocking the progression of CRPC and NEPC.

While CBX2 antagonism represents a promising therapeutic strategy, there are no inhibitors of CBX2 currently available. From a drug development standpoint, CBX2 possesses a chromodomain that binds H3K27me3 with high affinity and could be pharmacologically targeted. Adding value to this strategy, studies have shown that PRC1 complexes found at H3K27me3 sites were enriched in CBX2 compared to other CBX family members. To date, antagonists have been developed for a number of chromodomains, including that of CBX7. Since the chromodomains of CBX7 and CBX2 are largely conserved but display some structural differences, it is possible to synthesize small molecules with selectivity for CBX2. Thus, these compounds could disrupt the interaction between CBX2 and H3K27me3, providing a specific mechanism to inhibit CBX2 activity and reverse abnormal gene expression programs. In conclusion, this study provides the first evidence that the H3K27me3 reader CBX2 is functionally involved in any human cancer, thereby adding to the growing landscape of cancer epigenetics.

## Conclusions

There are currently no curative options for castration-resistant prostate cancer and thus there is a dire need to identify new potential therapeutic targets. We identified the polycomb group (PcG) member and epigenetic reader CBX2 as the most highly expressed PcG gene in metastatic and castration-resistant prostate cancers. Elevated expression correlated with aggressive disease and poor clinical outcomes. Functional analysis revealed that CBX2 is critical for prostate cancer cell survival. Our work positions CBX2 as a novel potential therapeutic target in CRPC.

## Methods

### Patient-derived xenograft models

As previously reported, the Living Tumor Lab (LTL, www.livingtumorlab.com) has developed a collection of high-fidelity PDXs implanted into the subrenal capsule of NOD-SCID mice [29]. We used the LTL313B/LTL313H model to investigate the role of CBX2 in metastasis and the LTL313B/BR model to assess the implications of CBX2 in drug-resistant CRPC [29]. Tumor tissues were obtained from patients through a protocol approved by the Clinical Research Ethics Board of the University of British Columbia (UBC) and the BC Cancer Agency (BCCA). All patients signed a consent form approved by the Ethics Board (UBC Ethics Board #: H09-01628 and H04-60131; VCHRI #: V09-0320 and V07-0058). Animal care and experimental procedures were carried out in accordance with the guidelines of the Canadian Council of Animal Care (CCAC) under the approval of the Animal Care Committee of University of British Columbia (permit #: A10-0100). The microarray gene expression data for these tumor lines have been previously deposited in the NCBI Gene Expression Omnibus (GEO) and are freely available under the accession number GSE41193.

### Bioinformatic database analysis

The Oncomine database was used to compare the expression of CBX2 between metastatic and non-metastatic PCa [30]. Data was acquired in an unbiased fashion by compiling all the Oncomine studies with significantly altered CBX2 expression (*p* ≤ 0.05). The cBIO portal (http://www.cbioportal.org/) was used to assess the genomic alterations affecting the CBX2 locus in PCa. In addition, the MSKCC dataset [31] was extracted from cBIO portal. Using this dataset, correlation between CBX2 and all other genes were calculated using the Pearson and Spearman correlation tests.

### Cell culture

All cell lines were maintained in RPMI 1640 growth medium (GIBCO) supplemented with 10 % fetal bovine serum (GIBCO) at 37 °C and 5 % CO_2_. For the androgen depletion experiment, LNCaP cells were initially plated in conditions described above for 24 h, following which media was changed to RPMI 1640 (GIBCO) supplemented with charcoal-stripped FBS (GIBCO), which has the property of being completely free of steroid hormones [40]. This charcoal-stripped media was then itself supplemented with DHT (10 nM) or not, and the cells were harvested at 6, 24, and 48 h after media change for qPCR analysis.

### qRT-PCR

RNA was extracted using the RNeasy Kit (Qiagen) according to the manufacturer’s protocol. NanoDrop technology (ND-1000, NanoDrop) was used to quantify extracted RNA, which was subsequently subjected to reverse transcription using the QuantiTect Kit (Qiagen). Quantification of cDNA was done using primers from IDT (see Table 6 for sequences) and SYBR Green Universal Master Mix (KAPA Biosystems) on an ABIPrism 7900HT platform (Applied Biosystems) as per the manufacturers’ instructions.

**Table 6:** qRT-PCR primers

Gene	Direction	Sequence (5′-3′)
CBX2	Forward	ATCGAGCACGTATTTGTCAC
CBX2	Reverse	AGTAATGCCTCAGGTTGAAG
CENPF	Forward	GAGGACCAACACCTGCTACC
CENPF	Reverse	GGCTAGTCTTTCCTGTCGGG
CEP55	Forward	CCGTTGTCTCTTCGATCGCT
CEP55	Reverse	GGCTTCGATCCCCACTTACT
DICER1	Forward	TGAAATGCTTGGCGACTCCT
DICER1	Reverse	GCCAATTCACAGGGGGATCA
FOXM1	Forward	ATAGCAAGCGAGTCCGCATT
FOXM1	Reverse	AGCAGCACTGATAAACAAAGAAAGA
HPRT1	Forward	GGTCAGGCAGTATAATCCAAAG
HPRT1	Reverse	CGATGTCAATAGGACTCCAGATG
INPP5A	Forward	TGTGACCGCATCCTCATGTC
INPP5A	Reverse	TGATTCGGAAGGCCAGGAAC
ITGB8	Forward	TTTGTCTGCCTGCAAAACGA
ITGB8	Reverse	GCACAGGATGCTGCATTTGA
MKI67	Forward	TGAGCCTGTACGGCTAAAACA
MKI67	Reverse	GGCCTTGGAATCTTGAGCTTT
PIK3R1	Forward	GATTCTCAGCAGCCAGCTCTGAT
PIK3R1	Reverse	GCAGGCTGTCGTTCATTCCAT
TERT	Forward	GAGAACAAGCTGTTTGCGGG
TERT	Reverse	AAGTTCACCACGCAGCCATA
TIMP2	Forward	GCGGTCAGTGAGAAGGAAGT
TIMP2	Reverse	GGAGGGGGCCGTGTAGATAA

### Western blot

Cell lysis was done using radioimmunoprecipitation assay (RIPA) buffer supplemented with a protease inhibitor cocktail (Roche). Bicinchoninic acid (BCA) protein assay (Thermo Fisher Scientific) was conducted to quantify protein concentrations in the resulting lysates. Fifteen micrograms of proteins were run on a 10 % sodium dodecyl sulfate polyacrylamide gel, transferred to a nitrocellulose membrane (Bio-Rad), and subjected to Western blot analysis. Primary rabbit antibodies specific to CBX2 (Thermo Fisher Scientific, Cat # PA5-30996, 1:1000) and actin (Thermo Fisher Scientific, Cat # PA1-16889, 1:4000) were incubated overnight at 4 °C, and goat anti-rabbit secondary antibody (Thermo Fisher Scientific, Cat # 31460, 1:15 000) was detected using electrochemiluminescence (ECL) kit (Thermo Fisher Scientific) according to the manufacturer’s protocol.

### Microscopy

Light microscopy images were obtained using the Axiovert 40 CFL (Zeiss) and the Axioplan 2 (Zeiss).

### siRNA knockdown

Twenty-four hours after seeding, cells at a confluency of 30–50 % were treated with 8 nm CBX2-specific or non-targeting siRNA (ON-TARGET plus siRNA, Dharmacon). Lipofectamine 2000 (Invitrogen) was used as the transfecting agent according to the manufacturer’s protocol, and cells were subjected to functional assays 24 to 120 h post-transfection.

### Caspase 3–7 activity

Seventy-two hours after CBX2 or non-targeting siRNA treatment in LNCaP and C4-2 (as described earlier), the relative caspase 3/7 activity was assessed using the Caspase-Glo 3/7 assay (Promega) according to the manufacturer’s protocol and chemiluminescence was measured with a spectrophotometer (Thermo Fisher Scientific).

### MTT analysis

At 1, 3, and 5 days post-treatment with siRNA, 3-(4,5-dimethylthiazol-2-yl)-2,5-diphenyltetrazolium bromide (MTT) solution (5 mg/ml, Sigma) was added to media and incubated for 3.5 h, after which, the cells were solubilized with dimethyl sulfoxide (DMSO) and absorbance was read at 570 nm using a spectrophotometer (Thermo Fisher Scientific).

### Microarray analysis

RNA was extracted from C4-2 cells treated with CBX2-specific or non-targeting siRNA 55 h post-treatment in triplicate, using the RNA isolation protocol described above in the qRT-PCR section. RNA quality was assessed using the Agilent 2100 Bioanalyzer. Samples were subjected to microarray analysis using the Agilent human GE 8x60 v1 array at the Laboratory for Advanced Genomic Analysis (LAGA) in Vancouver, BC. Differential gene expression was quantified using *T* test unpaired unequal variance (Welch), and *p* values were corrected for multiple testing using the Benjamini-Hochberg correction (*p* ≤ 0.05).

### Immunohistochemistry

The preparation of paraffin-embedded tissue sections and IHC were carried out as previously described [29, 41]. A CBX2-specific primary antibody was used (rabbit polyclonal, Pierce) and was recognized by a goat anti-rabbit secondary antibody (Vector Laboratory).

### Statistical analysis

Unsupervised hierarchical clustering and multivariate analysis of variance (MANOVA) were conducted using the R statistical package. Computational analyses of CBX2-regulated transcripts were carried out with the IPA software (Qiagen, June 2014 release). Unless otherwise mentioned, all analyses were done using *p* ≤ 0.05 (denoted as * in figures) as the significance threshold with the GraphPad Prism software (version 6).
